# CD4^+^ and CD8^+^ lymphocyte responses and cytokine profiles in visceral leishmaniasis patients following effective treatment in distinct transmission settings in Bihar, India

**DOI:** 10.3389/fimmu.2026.1919529

**Published:** 2026-10-22

**Authors:** Rahul Tiwari, Siddharth Sankar Singh, Sarita Singh, Vishal Kumar Singh, Rajneesh, Vibhav Gautam, Christian Engwerda, Shyam Sundar, Rajiv Kumar

**Affiliations:** 1 Centre of Experimental Medicine & Surgery, Institute of Medical Sciences, Banaras Hindu University, Varanasi, India; 2 Department of General Medicine, Institute of Medical Sciences, Banaras Hindu University, Varanasi, India; 3 QIMR Berghofer Medical Research Institute, Herston, QLD, Australia

**Keywords:** CD4^+^ lymphocytes, CD8^+^ lymphocytes, cytokine response, memory-phenotype, transmission, visceral leishmaniasis

## Abstract

Visceral leishmaniasis (VL), caused by *Leishmania donovani*, is characterized by a complex immune response, with a dominant Th1 profile crucial for parasite elimination. This study aimed to assess the persistence of immune memory and cytokine responses in individuals with a history of VL, focusing on the activation and proliferative capacity of CCR7/CD45RA defined CD4^+^ and CD8^+^ lymphocyte populations corresponding to central memory-like and effector memory-like phenotypes. We examined three distinct groups from areas with different transmission histories: areas with no transmission (NT), areas with continuous transmission (CT), and areas experiencing an outbreak (OB). Using soluble *Leishmania* antigen (SLA)-stimulated whole blood assays and flow cytometry, we measured Th1, Th2, and Th17 cytokine levels, activation marker CD69 and proliferation marker Ki67 in CD4^+^ and CD8^+^ lymphocytes. SLA stimulation elicited significant increases in IFN-γ, IL-10 and IL-2 relative to unstimulated controls within the NT and OB groups and was not significant in CT group. In contrast, comparison of antigen specific responses among transmission settings demonstrated significantly lower ΔIL-10 response in the CT group, whereas ΔIFN-γ and ΔIL-2 responses were not significantly different. CD4^+^CD45RA⁻CCR7^+^, CD4^+^CD45RA⁻CCR7⁻ CD8^+^CD45RA⁻CCR7^+^ and CD8^+^CD45RA⁻CCR7⁻ memory-phenotype lymphocyte subsets showed the higher absolute frequencies of activated cells in CT people, measured by expression of CD69, but not of proliferative responses. By contrast, the proliferation of CD4^+^CD45RA⁻CCR7^+^cells were increased in the OB group, underscoring variations in the functional nature of immunological memory between transmission circumstances. These findings indicate that while Th1 cytokine responses are preserved, the proliferative capacity of memory-phenotype lymphocytes varies by transmission setting, reflecting differences in antigen exposure and with implications for long-term protection and immune surveillance in VL elimination settings.

## 1 Introduction

Leishmaniasis encompasses a broad spectrum of clinical presentations, including cutaneous leishmaniasis (CL), visceral leishmaniasis (VL), and mucocutaneous leishmaniasis (MCL), caused by infection with protozoan parasites of the genera *Leishmania* and *Viannia* within the family *Trypanosomatidae*. These parasites are transmitted to humans primarily through the bite of infected female sand flies, which serve as biological vectors. The disease is closely associated with socioeconomic conditions, and most cases occur in regions lacking basic amenities. The World Health Organization (WHO) reports between 70,000 and 1 million cases of leishmaniasis annually, of which 50,000–90,000 are VL cases. However, this figure does not reflect the complete burden, as many cases go unreported. The WHO considers VL to have both outbreak and mortality potential, with only 25–45% of cases reported (https://www.who.int/news-room/fact-sheets/detail/leishmaniasis). In the Indian subcontinent, VL has historically been endemic in regions like Bihar, Uttar Pradesh, West Bengal, and Jharkhand. These areas, where VL transmission has been most prevalent, have experienced significant fluctuations in transmission rates over the years. Public health interventions, including improved diagnostics and vector control strategies, have led to a sharp decline in VL cases, with the number of reported cases in India dropping from over 40,000 in the early 1990s to under 1,000 annually in recent years (1). In India, only 508 VL and HIV–VL co-infection cases were reported in 2024 and 485 cases in 2025 (https://ncvbdc.mohfw.gov.in/index1.php?lang=1&level=2&sublinkid=5945&lid=3750).

However, despite these successes, outbreaks continue to occur sporadically, and regions once considered free of transmission are at risk of re-emergence, particularly if immunity in previously exposed populations wanes due to a lack of recurrent antigenic exposure. This highlights the ongoing need for continued surveillance, particularly in areas where VL has been historically prevalent but now reports low transmission.

Immunologically, VL is considered to be resolved by a Th1 dominant immune response where Interferon-gamma (IFN-γ)-producing CD4^+^ T cells play a central role (2), and active disease is associated with an insufficient Th1 response with the involvement of various pro- and anti-inflammatory cytokines like Interleukin (IL)-6, IL-12, IFN-γ, Tumor necrosis factor-alpha (TNF-α) with IL-10 and IL-4 [reviewed in (3–5)]. IFN-γ secretion by CD4^+^ T cells is essential for the intracellular elimination of *Leishmania* parasites within macrophages and dendritic cells. The critical role of IFN-γ in controlling VL is well-established and the level of serum IFN-γ is often related to disease control or progression (2, 6). Individuals with subclinical infection or asymptomatic individuals may also develop clinical disease if they fail to produce adequate IFN-γ (7) and studies in people with HIV-VL co-infection have shown a co-relationship between low IFN-γ levels and relapse following treatment completion (6). Furthermore, IL-10 is also associated with VL outcome, with increased IL-10 linked to active disease (8–11). Multiple studies have demonstrated an intricate relationship between IFN-γ and IL-10 in VL pathogenesis (12–14).

While CD4^+^ T cells are central to protective immunity in VL through IFN-γ–mediated macrophage activation, CD8^+^ T cells also contribute to anti-leishmanial immunity. Experimental and human studies have demonstrated that CD8^+^ T cells can produce IFN-γ and exert cytotoxic activity, supporting parasite control and shaping memory responses (15, 16). However, studies have reported CD8^+^ T cell exhaustion preventing their protective roles in humans (17). Moreover, CD8^+^ T cells may participate in long-term immune surveillance following exposure. Therefore, assessment of both CD4^+^ and CD8^+^ lymphocytes activation and proliferative responses can provide a more comprehensive evaluation of antigen-specific cellular immunity.

Individuals cured of VL are considered immune to the disease (14). In mouse models, it has been suggested that sterile cure doesn’t occurs and even after treatment some parasites persist providing concomitant immunity, while experimentally-induced complete cure results in loss of immunity rendering them susceptible to disease again (18–20). Long-term protection following infection and cure has been associated with the maintenance of memory T cell population, including central memory T cells (Tcm), effector memory T cells (Tem) and tissue resident memory T cells (Trm) (21, 22). However, most of the studies primarily concentrate on cutaneous leishmaniasis (CL). Studies on VL have also reported the development of protective Tcm and Tem cells that are able to secrete TNF-α and IFN-γ on antigenic stimulation *ex-vivo* (23, 24).

Given the crucial immunological role of memory cells, understanding their capacity for activation and proliferation is important. These properties are particularly relevant when investigating the cellular response to antigenic stimulation in chronic infections such as leishmaniasis. To unravel these dynamics, researchers have applied a variety of lymphoproliferation assays to evaluate functional outcomes. For example, several studies have employed approaches such as the measurement of Ki67, a marker of cellular proliferation (25–27). Moreover, assays measuring surface expression of CD69, an early activation marker, have been utilized to identify the activation status of memory T cells (25, 28–30). However, how this activation potential can change in areas of differing transmission and how long this memory can persist is a question to be addressed in context of VL.

Considering the idea that post-treatment immune memory persists in individuals, but a lack of exposure to antigens can render CD4^+^ and CD8^+^ memory lymphocytes ineffective in case of re-infection, the aim of this study was to investigate immune memory dynamics and cytokine production in post-treated VL individuals and establish how they vary with the endemicity of the disease in a given geographical location. We have used CD69 as a marker of activation and Ki67 as a marker of proliferation.

## 2 Materials and methods

### 2.1 Study participants selection

In this study, a total of 24 subjects (n=24) with a documented history of VL were recruited. All samples were collected in September 2023 from three geographically distinct regions of Muzaffarpur, Bihar, India. The process of sample collection was conducted at the Kala-azar Medical Research Centre (KAMRC), Muzaffarpur, Bihar. Each participant underwent a thorough medical examination to ensure the absence of any concurrent disease conditions. Clinical details for all the study participants are provided in **Table 1**. To explore the impact of different VL transmission backgrounds, participants were stratified into three groups: eight individuals from areas with no reported VL cases in the last five years (no cases after 2017) but cases were reported before that time (no transmission, NT), eight individuals from regions experiencing ongoing VL cases (continuous transmission, CT, since 2013 to 2022-23), and eight individuals from locations where VL had recently re-emerged after a disease-free interval (outbreak, OB, cases after year 2017). All enrolled participants in this study had a documented history of successfully treated VL and were sampled after treatment completion. The NT, OB, and CT classifications were based on the documented transmission history of the participant’s residential localities and therefore represent area-level epidemiological categories. Detailed individual-level information on duration of continuous residence, migration and travel to other VL-endemic areas was not systematically collected as part of the study, as in this rural endemic setting, accurately documenting all movements that might have resulted in exposure would be difficult in practice and subject to substantial recall uncertainty. Therefore, the possibility of exposure outside the assigned transmission setting (NT, OB and CT) cannot be completely excluded. Consequently, the transmission classifications should not be interpreted as direct measures of each participant’s cumulative exposure to *L. donovani*. This selection strategy was consequently used to examine area-level transmission setting and post-treatment immune responses and memory lymphocyte profiles, rather than to infer the individual cumulative exposure history of each participant.

**Table 1:** Clinical details.

Variables	No transmission (n=8)	Outbreak (n=8)	Continuous transmission (n=8)
*Age (in years), Mean +/- SD*	42 +/- 19.6	37.625 +/- 18.66	19.5 +/- 12.86
*Median*	40	42.5	13
*Male*	5	3	4
*Female*	3	5	4
*Previous VL history*	8 (100%)	8 (100%)	8 (100%)
*Successfully treated VL*	8 (100%)	8 (100%)	8 (100%)
*HIV status*	Negative	Negative	Negative

### 2.2 Preparation of soluble leishmania antigen, whole blood assay and cytokine measurement

Soluble *Leishmania* antigen (SLA) was prepared from *Leishmania donovani* clinical isolates obtained at KAMRC, Muzaffarpur, Bihar, India, as previously described by Gidwani et al. (31). Briefly, parasites derived from clinical isolates were cultured in Medium 199 until transformation to promastigotes, and stationary-phase promastigotes were harvested by centrifugation. The parasite pellet was washed three times with cold PBS and resuspended in lysis buffer containing 10 mM Tris-HCl, 1 mM EDTA, 1.6 mM phenylmethanesulfonyl fluoride (PMSF), and 50 µg/mL leupeptin. Parasites were disrupted by repeated sonication, followed by centrifugation at 4 °C. After removal of the lipid layer, the supernatant was ultracentrifuged at 100,000 × g for 4 hours at 4 °C. The resulting supernatant was dialysed overnight against PBS and stored at −80 °C until use. Protein concentration was determined using a Micro BCA Protein Assay Kit (Thermo Scientific). SLA was used at a final protein concentration of 10 µg/mL in the whole-blood stimulation assay. Whole blood assays (WBA) were performed using 3 mL of heparinized peripheral blood collected from each participant (n=24). Plasma was removed to eliminate pre-existing circulating cytokines, and the blood was washed with 1x PBS. The original blood volume was subsequently restored by replacing the plasma with HI-FBS. The reconstituted blood was aliquoted into three polypropylene culture tubes (1 mL per tube) and stimulated with PBS as an unstimulated control, SLA, and PHA. PHA stimulation was included as a positive control to verify the overall functional responsiveness and the performance of the assay. Following stimulation, cultures were incubated for 24 hrs at 37 °C in a humidified atmosphere containing 5% CO_2_. After incubation, culture supernatants were harvested, and cytokine concentrations were quantified using a Th1/Th2/Th17 cytometric bead array (CBA) kit (BD Biosciences), according to the manufacturer’s instructions (for list of reagents see **Supplementary Table S1**).

### 2.3 PBMC stimulation for flow cytometric analysis

Peripheral blood (∼8ml) was collected in heparinised tubes and 4ml of blood was used to isolate peripheral blood mononuclear cells (PBMCs) using Lymphoprep (STEMCELL Technologies) a density gradient medium. Following isolation, cells were counted using a haemocytometer. For each subject, 1 × 10^6^ PBMCs were distributed into three separate culture tubes, corresponding to the three stimulation conditions. Cells were resuspended in 1 mL of RPMI-1640 medium supplemented with 10% heat-inactivated fetal bovine serum (HI-FBS) (Gibco, life technologies, USA), 10,000 U/mL penicillin, and 10,000 μg/mL streptomycin. The cultures were then stimulated with SLA at a final concentration of 10 µg/mL, anti-human CD3/CD28 antibodies (positive control), or phosphate-buffered saline (PBS; negative control). Anti-human CD3 antibody was used at a final concentration of 1 μg/mL, and anti-human CD28 antibody was used at a final concentration of 5 μg/mL. Anti-CD3/CD28 stimulation was used as a polyclonal positive control to verify the functional responsiveness of lymphocytes independently of antigen-specific response. PBS-treated cells are hereafter referred to as “NIL” in subsequent sections. After stimulation, the cells were incubated for 72 hours in an incubator at 37 °Celsius with 5% CO_2_.

### 2.4 Flow cytometry

After a 72-hour *in vitro* culture, the culture supernatant was carefully collected, and cells were washed once with phosphate-buffered saline (PBS). The cell suspension was then centrifuged to pellet the cells, which were subsequently resuspended in fresh PBS for further processing. For immunophenotyping, harvested cells were stained with a titrated panel of fluorochrome-conjugated monoclonal antibodies targeting cell surface and intracellular markers of interest. Prior to fixation and permeabilization, cells were stained with Zombie Aqua viability dye (BioLegend) to exclude dead cells from analysis. Lymphocyte subsets were delineated according to the differential expression of CD45RA and CCR7. Subsets were classified into CD45RA^−^CCR7^+^, CD45RA^−^CCR7^−^ and CD45RA^+^CCR7^−^ memory-phenotype lymphocytes. The activation status of these subsets was determined by CD69 expression, and proliferative capacity was assessed by intracellular staining for Ki67. Following staining, cells were acquired on an LSRFortessa flow cytometer (BD Biosciences) using standardized instrument settings. All samples were compensated using single-stained controls. Flow cytometric data was analysed using FlowJo (version 10.10, BD Biosciences). We gated lymphocytes by using forward scatter (FSC) and side scatter (SSC) properties, followed by single-cell discrimination and live cells by the help of Zombie Aqua viability dye. Gating of CD4^+^ or CD8^+^ lymphocytes was performed using specific markers, and memory-phenotype showing subsets were identified based on CD45RA and CCR7 expression (**Supplementary Figure S1A**). Owing to reagent limitations, CD3 was not included in the final staining panel. Fluorescence-minus-one (FMO) controls were used to determine the positive gate for CD69, Ki67, CCR7 and CD45RA and identical gating strategies were applied consistently across all samples (for list of flow cytometry reagents see **Supplementary Table S1**).

### 2.5 Statistical analysis

Cytokine responses were analysed using non-parametric tests due to non-Gaussian distribution. Paired comparisons were performed using the Wilcoxon signed-rank test. For cytokine analyses, outlier assessment was performed using the ROUT (robust regression and outlier removal) method implemented in GraphPad Prism. One participant in the NT group was identified as an outlier and was excluded from the cytokine analyses; therefore, analyses were performed using NT (n=7), OB (n=8), and CT (n=8). Between-group comparisons of Δ (SLA–NIL) responses were performed using the Kruskal–Wallis test followed by Dunn’s multiple comparison correction. A two-sided p < 0.05 was considered significant. All these analyses were performed using GraphPad PRISM (Version 8).

Flow cytometry data were analysed based on the percentage frequency of cells within the predefined populations. Paired comparisons between unstimulated control and SLA stimulation within the same individuals were performed using the Wilcoxon signed-rank test. Comparisons involving anti-CD3/CD28 stimulation as a positive control were also performed using paired statistical analysis. Differences in antigen-specific responses between NT, OB, and CT groups were analysed using the Kruskal–Wallis test followed by Dunn’s multiple comparison correction. All statistical analyses were performed using GraphPad Prism (version 8) and p-values <0.05 were considered statistically significant.

## 3 Results

### 3.1 Differential modulation of antigen-specific cytokine recall responses across transmission settings

Whole blood stimulation with SLA elicited antigen-specific cytokine responses that differed according to transmission background. Following stimulation, cells were assessed by flow cytometry, where CD4^+^ and CD8^+^ lymphocytes were sub-gated into CD45RA^+^ CCR7^+^ naïve lymphocytes and memory-phenotype showing lymphocytes like CD45RA^+^ CCR7^-^, CD45RA^-^ CCR7^-^ and CD45RA^-^ CCR7^+^ cells (**Supplementary Figure S1A**). In NT and OB groups, paired analysis demonstrated significant induction of IFN-γ, IL-10 and IL-2, compared to NIL (no antigen) controls, indicating preserved antigen-specific responsiveness in these settings (**Figure 1**). In contrast, individuals from CT areas exhibited comparatively attenuated responses, with none of these cytokines reaching statistical significance in paired comparisons (**Figure 1**). TNF-α and IL-17 were not significantly elevated in any group, suggesting that SLA-driven recall responses in this cohort were characterized predominantly by Th1/regulatory cell dynamics rather than inflammatory or Th17-skewed activation (**Supplementary Figures S2A–F**).

**Figure 1:**
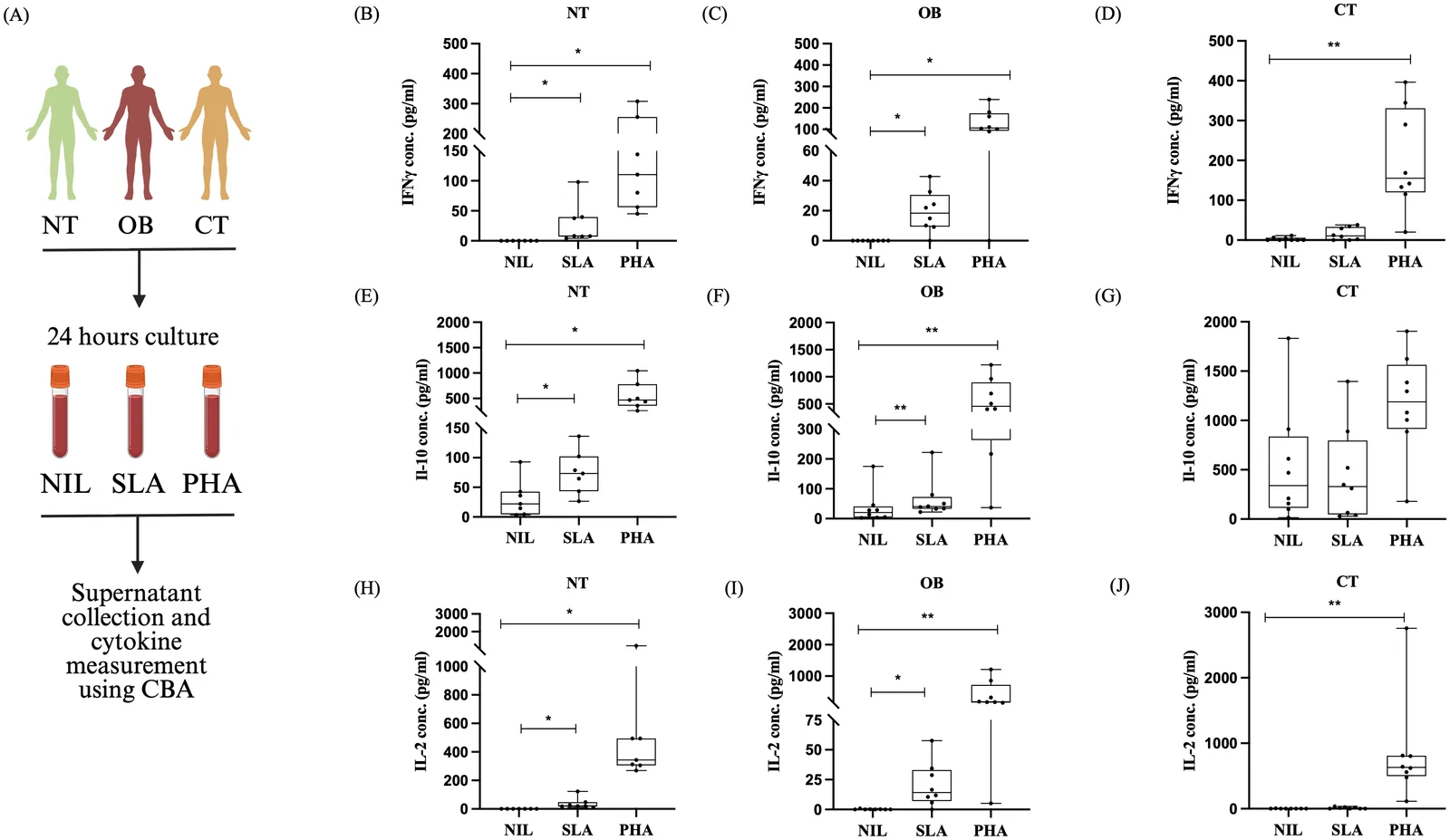
Antigen-specific cytokine recall responses following *Leishmania* specific antigen stimulation. Experimental scheme of whole blood stimulation and cytokine analysis **(A)**. Whole blood samples were stimulated with SLA and PHA and cytokine production was compared with unstimulated control (NIL) within the same individuals. IFN-γ responses in NT (n= 7), OB (n= 8), and CT (n= 8) groups are shown in **(B–D)**, IL-10 responses in **(E–G)**, and IL-2 responses in **(H–J)**. Statistical comparisons between NIL, SLA and PHA conditions were performed using the Wilcoxon signed-rank test. Cytokine concentrations are expressed as pg/ml. NT, no transmission; OB, outbreak; CT, continuous transmission; SLA, soluble *Leishmania* antigen; NIL, unstimulated control; PHA, Phytohemagglutinin. Statistical significance was defined as *p < 0.05 and **p < 0.01. [Experimental scheme was created using BioRender.com].

To determine whether transmission setting influenced the magnitude of antigen-specific responses, Δ cytokine values (SLA–NIL) were compared across all groups. ΔIFN-γ did not differ significantly between groups (p = 0.4527) (**Figure 2A**). In contrast, significant differences were observed for ΔIL-10 (p = 0.0180; adjusted p = 0.0149) (**Figure 2B**). IL-10 plays a critical regulatory role in human VL by modulating effector responses and preventing excessive immunopathology. ΔIL-2 showed a non-significant trend across groups (p = 0.0819), with numerically higher responses in NT compared to CT, but without statistically significant pairwise differences following adjustment (**Figure 2C**). IL-2 is essential for T-cell proliferation and maintenance of antigen-specific memory. Although suggestive of reduced proliferative recall in CT, the present dataset does not support a statistically robust transmission effect for IL-2 after outlier exclusion.

**Figure 2:**
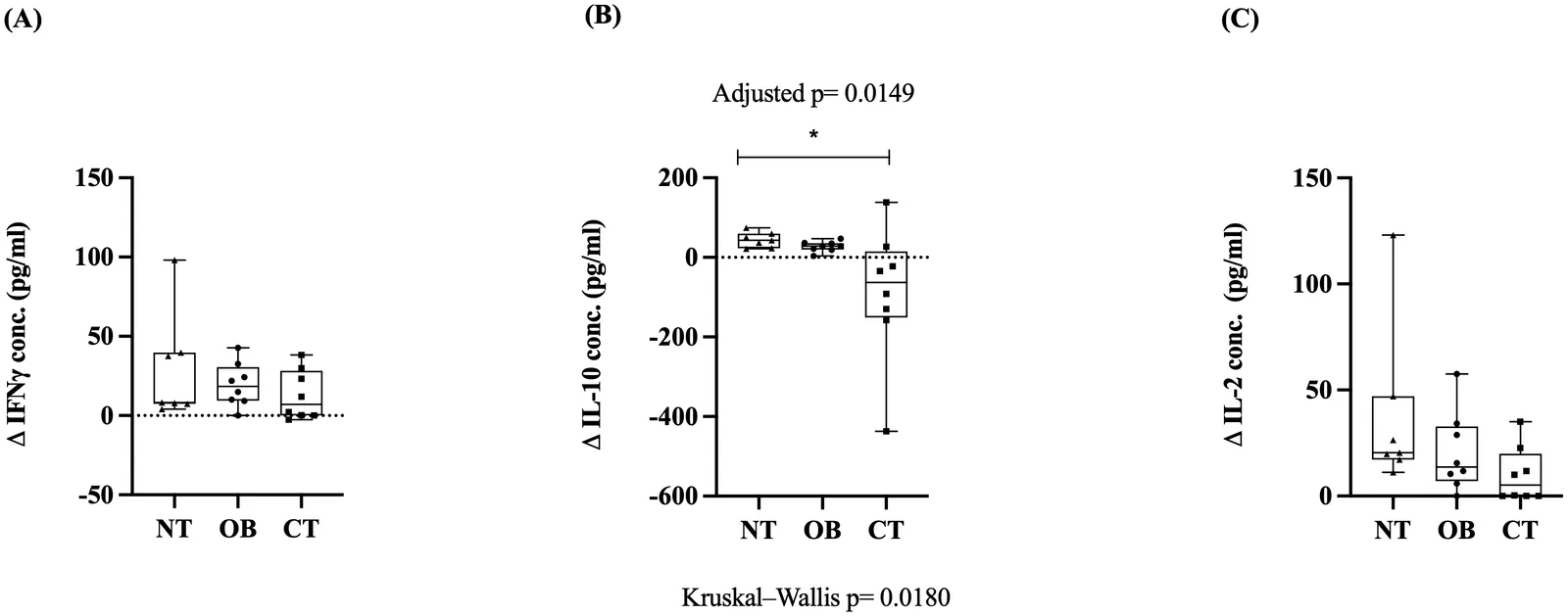
Comparison of antigen-specific cytokine recall responses across transmission settings. ΔIFN-γ **(A)**, ΔIL-10 **(B)**, and ΔIL-2 **(C)** responses in individuals from NT (n = 7), OB (n = 8), and CT (n = 8) groups. Responses were calculated as the difference between SLA-stimulated and unstimulated control (NIL) cytokine concentrations (Δ = SLA − NIL). Cytokine concentrations are expressed as pg/ml. NT, no transmission; OB, outbreak; CT, continuous transmission; SLA, soluble *Leishmania* antigen; NIL, unstimulated control; Between-group comparisons of antigen-specific cytokine responses were performed using the Kruskal–Wallis test followed by Dunn’s multiple comparison correction. The overall Kruskal–Wallis p-values and significant adjusted pairwise comparisons are indicated in the graphs where significance was found. Statistical significance was defined as *p < 0.05 and **p < 0.01.

ΔIL-17 and ΔTNF-α did not differ significantly across groups, consistent with the absence of transmission-associated shifts in inflammatory or Th17-type recall responses (**Supplementary Figures S3A, B**).

Collectively, these findings demonstrate transmission-associated differences in antigen-specific cytokine recall responses, whereby ΔIL-10 was significantly reduced in CT compared with NT, while IFN-γ and IL-2 responses showed limited antigen-specific changes.

### 3.2 Antigen-induced activation of CD4^+^ and CD8^+^ memory phenotype lymphocytes

To evaluate antigen-specific activation, whole blood samples were stimulated with SLA, and CD69 expression was assessed within CD45RA^+^CCR7^+^ naïve lymphocytes and CD45RA⁻CCR7^+^/CD45RA⁻CCR7⁻ memory-phenotype lymphocyte subsets (see **Supplementary Figure S1A** for gating strategy).

SLA stimulation did not significantly alter total CD4^+^ lymphocytes frequencies in NT (p = 0.6875), OB (p = 0.3125), or CT (p = 0.0781), indicating that antigen exposure did not affect overall CD4^+^ lymphocytes abundance (**Supplementary Figure S1B**). Instead, activation was predominantly observed within memory like compartments. CD69 expression was significantly upregulated in CD4^+^ CD45RA⁻CCR7^+^ lymphocytes in NT (p = 0.0078), OB (p = 0.0156), and CT (p = 0.0156) (**Figures 3A–C**), and similarly increased in CD4^+^CCR7⁻CD45RA⁻ lymphocytes across all three settings (NT p = 0.0078; OB p = 0.0078; CT p = 0.0078) (**Figures 4A–C**), demonstrating preserved activation in all the groups, unlike IFN-γ response that was only preserved in NT and OB groups (A representative bivariate plot with heatmap statistic comparing CD4^+^CD45RA^−^CCR7^+^, CD4^+^CD45RA^−^CCR7^−^, CD8^+^CD45RA^−^CCR7^+^ and CD8^+^CD45RA^−^CCR7^−^ memory-phenotype lymphocytes against CD69 have been shown in **Supplementary Figures S4A–D**).

**Figure 3:**
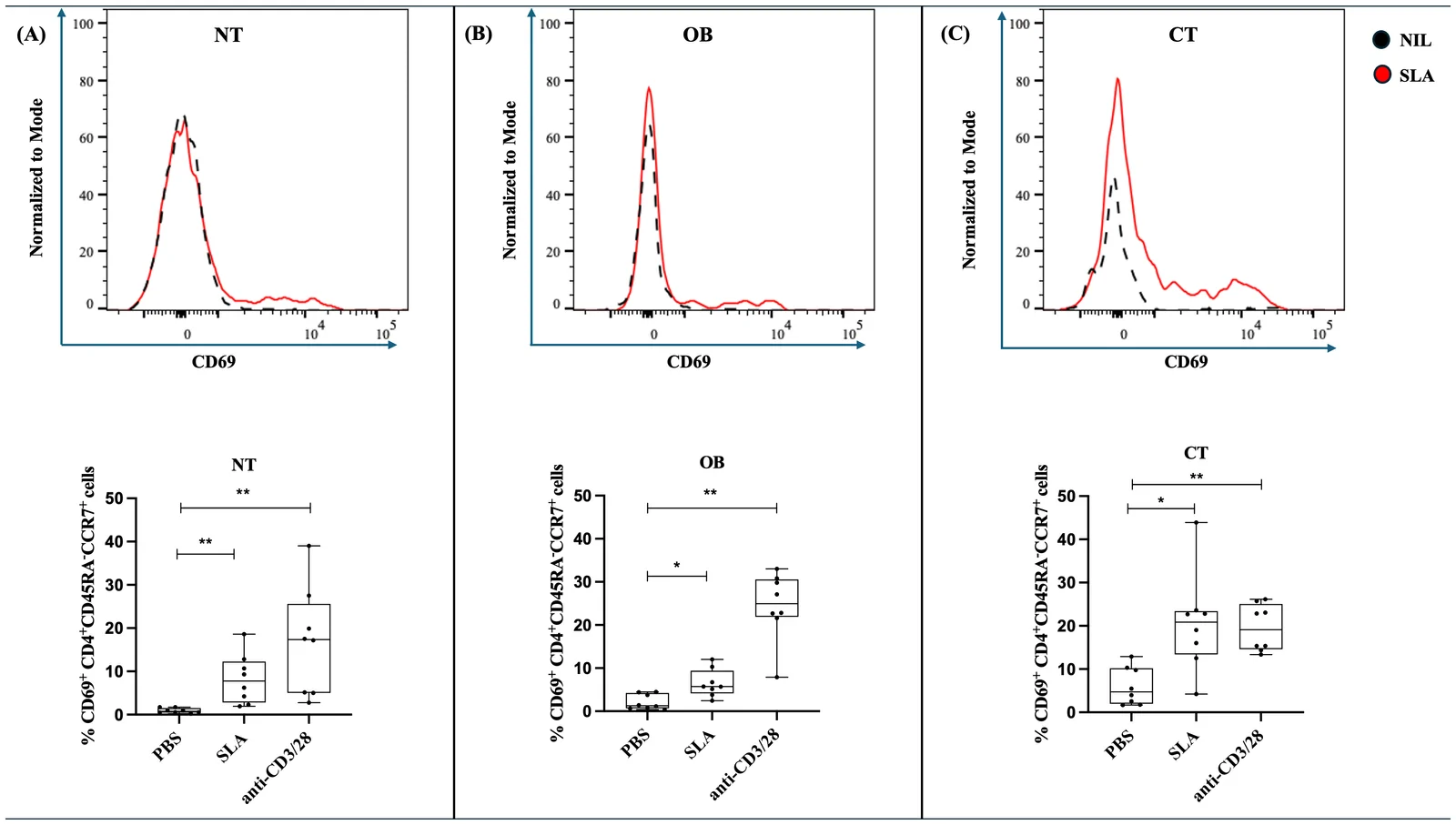
Antigen-induced activation of CD4^+^CD45RA^−^CCR7^+^ lymphocytes. CD69 expression was assessed within CD4^+^CD45RA^−^CCR7^+^ lymphocyte populations following stimulation with PBS, SLA and anti-CD3/CD28 in individuals from NT (n= 8), OB (n= 8), and CT (n= 8) groups **(A–C)**. PBS and anti-CD3/CD28 stimulation were included as negative and positive control respectively. Data represent the percentage frequency of CD69^+^ cells within CD4^+^ lymphocytes. Paired comparisons between stimulation conditions within each individual were performed using the Wilcoxon signed-rank test. NT, no transmission; OB, outbreak; CT, continuous transmission; PBS, Phosphate buffer saline;SLA, soluble *Leishmania* antigen. Statistical significance was defined as *p < 0.05 and **p < 0.01.

**Figure 4:**
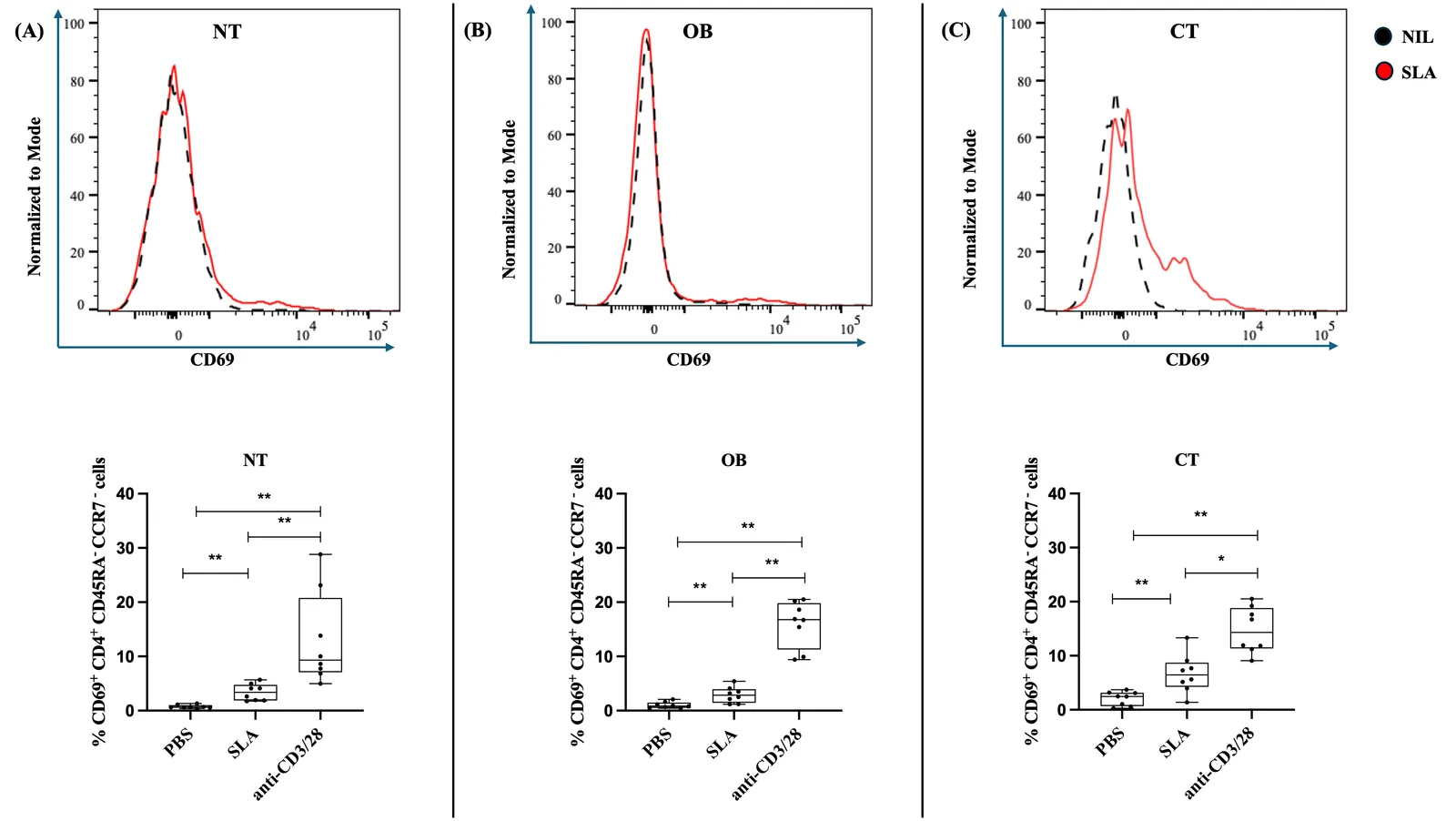
Antigen-induced activation of CD4^+^CD45RA^−^CCR7^−^ lymphocytes. CD69 expression was assessed within CD4^+^CD45RA^−^CCR7^−^ populations following stimulation with PBS, SLA, and anti-CD3/CD28 in individuals from NT (n= 8), OB (n= 8), and CT (n= 8) groups **(A–C)**. PBS and anti-CD3/CD28 stimulation were included as negative and positive controls, respectively. Data represent the percentage frequency of CD69^+^ cells within CD4^+^CD45RA^−^CCR7^−^ populations. Paired comparisons between stimulation conditions within individuals were performed using the Wilcoxon signed-rank test. NT, no transmission; OB, outbreak; CT, continuous transmission; PBS, phosphate-buffered saline; SLA, soluble *Leishmania* antigen. Statistical significance was defined as *p < 0.05 and **p < 0.01.

A comparison of SLA-stimulated frequencies between groups showed significant differences in CD69^+^ CD4^+^CD45RA^−^CCR7^+^ memory-phenotype lymphocytes (Kruskal–Wallis p = 0.0087). CT had higher activation than NT (adjusted p = 0.0400) and OB (adjusted p = 0.0140) (**Figure 5A**). CD69^+^ CD4^+^CD45RA^−^CCR7^−^ memory-phenotype lymphocytes also differed across groups (p = 0.0341), driven by higher activation in CT compared with OB (adjusted p = 0.0362) (**Figure 5B**). These findings indicate that while antigen-induced activation is present across all transmission settings, individuals from continuous transmission areas display enhanced memory activation magnitude, although this wasn’t reflected in cytokine production.

**Figure 5:**
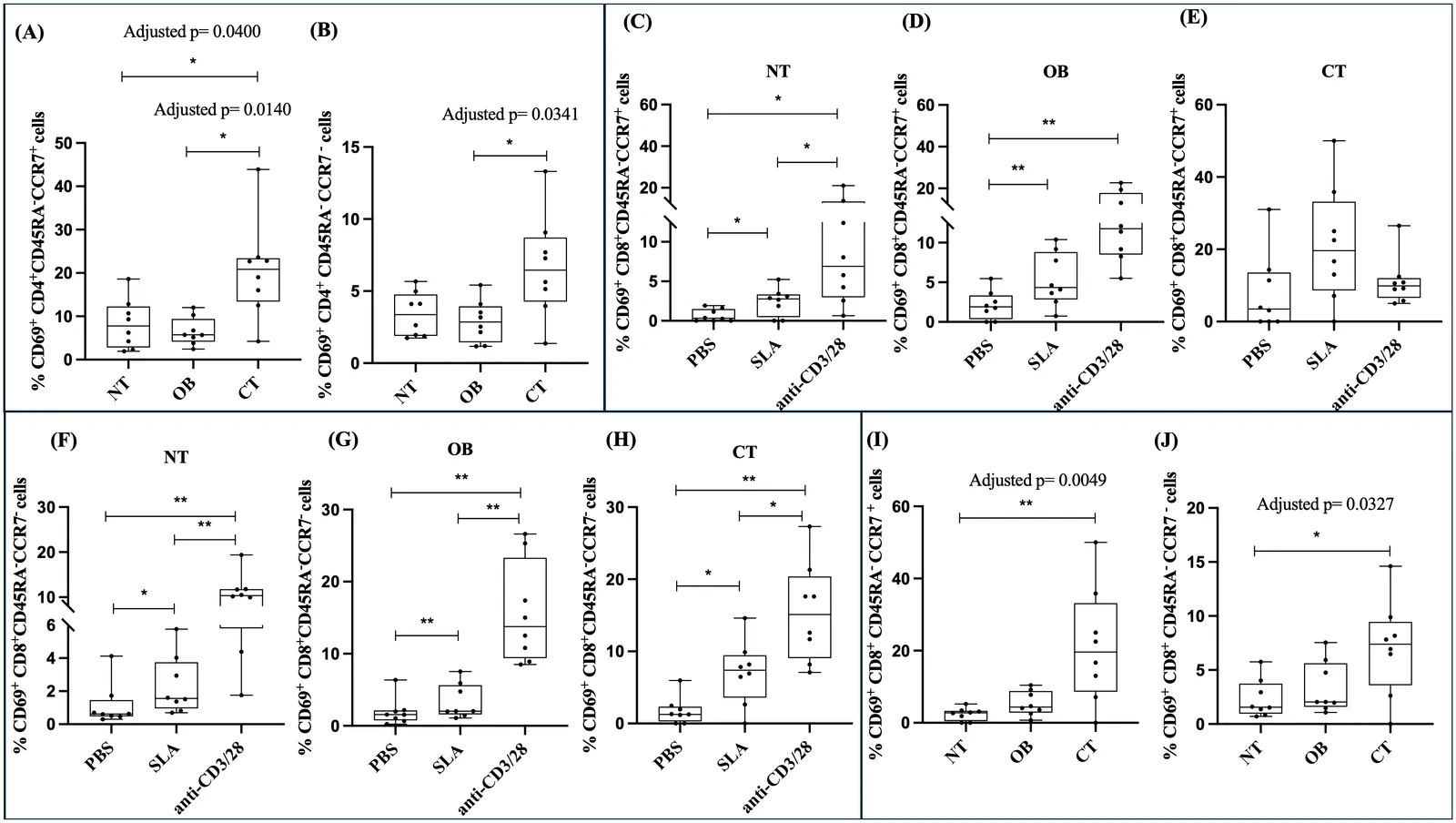
Differential activation of CD4^+^ and CD8^+^ CCR7/CD45RA-defined lymphocyte populations across transmission settings. Between-group comparison of SLA-induced CD69 expression within CD4^+^CD45RA^−^CCR7^+^ **(A)** and CD4^+^CD45RA^−^CCR7^−^ memory-phenotype lymphocytes **(B)** across NT, OB, and CT groups respectively. Antigen-specific activation of CD8^+^CD45RA^−^CCR7^+^ **(C–E)** and CD8^+^CD45RA^−^CCR7^−^ memory-phenotype lymphocytes following stimulation with PBS, SLA and anti-CD3/CD28 **(F–H)**. PBS and anti-CD3/CD28 stimulation were included as negative and positive controls, respectively, to assess baseline and maximal activation responses. SLA-induced CD69^+^ CD8^+^CD45RA^−^CCR7^+^ and CD69^+^ CD8^+^CD45RA^−^CCR7^−^ lymphocytes frequencies across NT, OB, and CT groups respectively **(I, J)**. Between-group comparisons were performed using the Kruskal–Wallis test followed by Dunn’s multiple comparison correction. Within-group paired comparisons between stimulation conditions were performed using the Wilcoxon signed-rank test. NT, no transmission; OB, outbreak; CT, continuous transmission; PBS, phosphate-buffered saline; SLA, soluble *Leishmania* antigen. Statistical significance was defined as *p < 0.05 and **p < 0.01.

Within the CD8 compartment, total CD8^+^ lymphocyte frequencies were not significantly altered by SLA stimulation (**Supplementary Figures S3C–E**). However, subset-specific activation was observed. CD69^+^ CD8^+^CD45RA^−^CCR7^+^ lymphocytes frequency increased in NT (p = 0.0312), and OB (p = 0.0078), and remained insignificant in CT group (p = 0.2422), whereas CD69^+^ CD8^+^CD45RA^−^CCR7^−^lymphocytes increased in NT (p = 0.0156), OB (p = 0.0078), and CT (p = 0.0156) (**Figures 5C–H**). When comparing activation between groups, following SLA stimulation CD8^+^CD45RA^−^CCR7^+^ lymphocytes showed a significant overall difference among NT, OB, and CT groups (Kruskal–Wallis p = 0.0069). Dunn’s multiple comparison analysis revealed significantly higher activation of CD8^+^CD45RA^−^CCR7^+^ lymphocytes in the CT group compared with the NT group (adjusted p = 0.0049), whereas no significant differences were observed between NT and OB or between OB and CT groups (**Figure 5I**).

For CD8^+^CD45RA^−^CCR7^−^ memory-phenotype lymphocytes a significant overall difference in activation was observed among NT, OB and CT groups (Kruskal-Wallis p = 0.0347). Dunn’s multiple comparison analysis revealed significantly higher activation of CD8^+^CD45RA^−^CCR7^−^ lymphocytes in the CT group compared with the NT group (adjusted p = 0.0327). No significant differences were observed between NT and OB or between OB and CT groups memory-phenotype lymphocytes (**Figure 5J**).

To determine whether differences in SLA-induced CD69 expression reflected antigen-specific activation rather than variation in baseline activation states, CD69 responses were further normalized to unstimulated controls by calculating ΔCD69 (SLA−PBS) for each individual. Following normalization, no significant differences were observed among NT, OB, and CT groups for CD4^+^CD45RA^−^CCR7^+^ cells, CD4^+^CD45RA^−^CCR7^−^ cells, or CD8^+^ CD45RA^−^CCR7^+^ cells (**Supplementary Figures S3F–H**). In contrast, ΔCD69 expression in CD8^+^CD45RA^−^CCR7^−^ lymphocyte populations showed a significant overall difference among transmission groups (Kruskal–Wallis p = 0.0142). Dunn’s multiple comparison analysis demonstrated higher antigen-induced activation in CD8^+^CD45RA^−^CCR7^−^ lymphocytes in CT group compared with NT (adjusted p = 0.0266) and OB (adjusted p = 0.0463), whereas no significant difference was observed between NT and OB groups (**Supplementary Figure S3I**). These findings indicate that continuous transmission is associated with a selective increase in antigen-induced CD8^+^CD45RA^−^CCR7^−^ lymphocyte activation.

### 3.3 Proliferative capacity of activated CD4^+^ and CD8^+^ lymphocytes

To determine whether antigen-driven activation translated into proliferative expansion, Ki67 expression was evaluated within CD69^+^ subsets. CD69^+^Ki67^+^ expressing CD4^+^CD45RA^−^CCR7^+^ lymphocytes showed a significant increase following SLA stimulation compared with PBS/NIL controls in the OB group (p = 0.0312), whereas no significant induction was observed in NT or CT groups (**Figures 6A–C**). In addition, anti-CD3/CD28 stimulation induced significant proliferation in CD4^+^CD45RA^−^CCR7^+^ cells in OB and CT groups compared with PBS controls, confirming the CD4^+^ proliferative capacity of these cells following strong polyclonal stimulation. Similar analysis of CD4^+^CD45RA^−^CCR7^−^ cells showed significant induction following anti-CD3/CD28 stimulation compared with PBS controls in NT and OB groups, whereas this difference was not observed in CT (**Figures 6D–F**). Together, these findings demonstrate that while lymphocytes showing memory-phenotype remain capable of proliferation following strong stimulation, SLA-driven proliferative responses are more restricted and vary according to transmission setting and lymphocyte subset (A representative bivalent plot with heatmap statistic comparing CD69^+^ CD4^+^CD45RA^−^CCR7^+^ and CD4^+^CD45RA^−^CCR7^−^ lymphocytes against Ki67 is shown in **Supplementary Figures S5A, B**).

**Figure 6:**
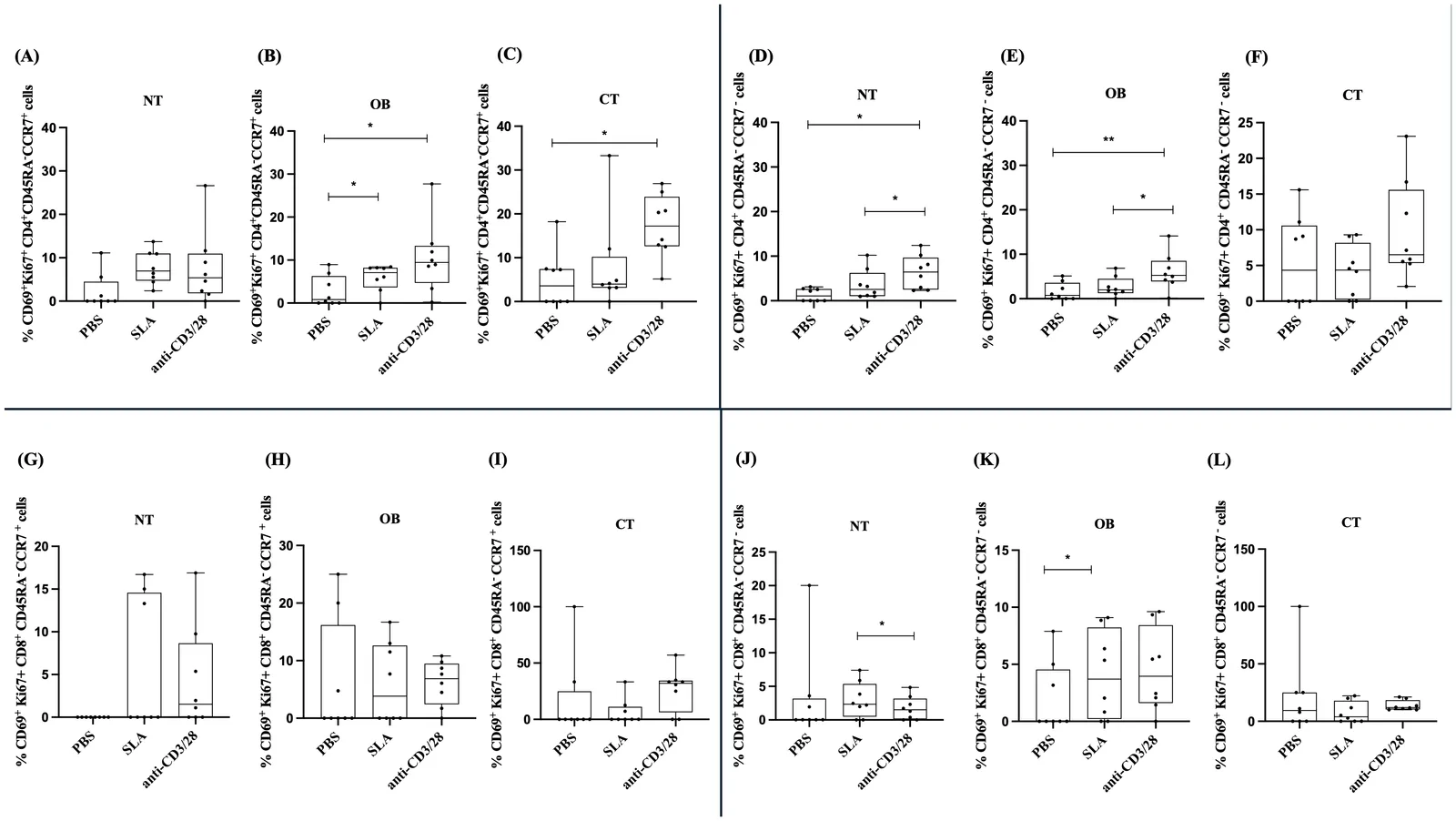
Proliferative responses of activated CCR7/CD45RA-defined lymphocyte populations. Ki67-associated proliferative responses were assessed within CD69^+^ activated memory phenotype-associated lymphocyte populations following PBS, SLA and anti-CD3/CD28 stimulation. The frequency of CD69^+^Ki67^+^ double-positive cells was evaluated in CD4^+^CD45RA^−^CCR7^+^ **(A–C)**, CD4^+^CD45RA^−^CCR7^−^ **(D–F)**, CD8^+^CD45RA^−^CCR7^+^ **(G–I)**, and CD8^+^CD45RA^−^CCR7^−^ **(J–L)** memory-phenotype lymphocytes from individuals in the NT, OB and CT groups. Within each transmission setting, comparisons between PBS control, SLA stimulation and anti-CD3/CD28 stimulation were performed using the Wilcoxon signed-rank test. CD69^+^Ki67^+^ cells represent activated memory-phenotype lymphocytes undergoing proliferative expansion following stimulation. NT, no transmission; OB, outbreak; CT, continuous transmission; PBS, phosphate-buffered saline; SLA, soluble *Leishmania* antigen. Statistical significance was defined as *p < 0.05 and **p < 0.01.

In the CD8 compartment, Ki67-associated proliferative responses were more heterogeneous compared with CD69-defined activation. Within the CD8^+^ compartment, SLA stimulation induced a significant increase in CD69^+^Ki67^+^ CD8^+^CD45RA⁻CCR7⁻ lymphocytes in the OB group compared with PBS controls. In contrast, no significant SLA-induced proliferative response was observed in the other analysed CD8^+^ CCR7/CD45RA-defined populations or transmission groups (**Figures 6G–L**). Together, these findings indicate that antigen recall induces activation across lymphocytes memory compartments, whereas proliferative responses are more variable and depend on both the transmission setting and specific lymphocyte subsets.

## 4 Discussion

This study uniquely compares antigen-specific recall responses across distinct epidemiological settings (NT, OB, CT), providing a brief insight into how transmission intensity is associated with immune memory maintenance following VL cure. Such heterogeneity in post-treatment immune responses has been reported in human VL cohorts, where antigen-specific cytokine recall varies depending on exposure history and endemicity (12).

We observed that stimulation of whole blood with SLA resulted in significant increases in IFN-γ, IL-2, and IL-10 in individuals from NT and OB groups. In contrast, individuals from CT group did not exhibit significant antigen-specific induction of IFN-γ or other measured cytokines following SLA stimulation. These cytokines are critical for controlling VL, with IFN-γ having a well-established role in macrophage activation and parasite clearance. In human VL, restoration of IFN-γ together with persistent IL-10 has been shown to characterize post-treatment immune responses, highlighting the dual role of effector and regulatory pathways (2, 14). IL-10-mediated suppression of anti-leishmanial immunity has been well demonstrated in human splenic aspirate studies (9).

Altered cytokine recall responses associated with continuing transmission in CT were characterized by reduced IL-10 responses together with impaired antigen-specific IFN-γ and IL-2 recall. Importantly, these changes were associated with a lack of substantial antigen-specific IFN-γ production, suggesting that immunity is defined by qualitative changes, not selective maintenance of Th1 effector responses.

This pattern may reflect a re-calibration of the cytokine milieu due to repeated antigen exposure. In endemic VL settings, repetitive exposure, has also been shown to modify cytokine profiles in a similar manner (12).

To further characterize antigen-responsive CCR7/CD45RA-defined lymphocyte, we assessed activation-associated CD69 expression within CD4^+^ and CD8^+^ memory compartments following SLA stimulation. SLA stimulation induced significant change in CD69 expression across these lymphocytes, indicating preserved antigen responsiveness after VL treatment. However, because CD69 reflects activation rather than functional capacity, these findings were interpreted together with cytokine recall and proliferative responses. Human studies have demonstrated that cured VL individuals retain multifunctional CD4^+^ and CD8^+^ memory T cells capable of producing IFN-γ and TNF-α upon antigenic stimulation (23). Although absolute CD69 frequencies following SLA stimulation were higher in CT individuals, baseline-normalized analysis (ΔCD69: SLA−PBS) demonstrated that these differences were not maintained across all subsets of memory like lymphocytes and only CD8^+^CD45RA^−^CCR7^−^ cells retained higher antigen-induced activation in CT compared with NT and OB groups.

Interestingly, activation-associated CD69 expression and proliferative responses did not show a uniform relationship across transmission settings. Although CT individuals retained increased CD8^+^CD45RA^−^CCR7^−^ activation after baseline normalization, this was not accompanied by broad enhancement of Ki67-associated proliferation. In contrast, CD8^+^CD45RA^−^CCR7^−^ cells from the OB group demonstrated significant antigen-induced proliferative responses following SLA stimulation, suggesting preserved proliferative recall capacity in this setting. These findings indicate that activation and proliferative expansion of memory-phenotype associated lymphocyte represent distinct functional properties of immune responses. This phenomenon is somewhat consistent with observations in chronic infections where persistent antigen exposure leads to sustained activation with reduced proliferative renewal (32).

The role of IL-10 in VL pathogenesis is complex. While IL-10 is essential for limiting immunopathology during infection, high levels of IL-10 have been associated with disease progression and impaired immunity. Higher IL-10 responses in NT and OB may reflect preserved antigen-responsive regulatory pathways following parasite clearance, whereas reduced IL-10 in CT may indicate altered immune regulation under conditions of continuous antigen exposure. The central role of IL-10 in regulating disease progression and immune suppression in VL is well established (8).

Taken together, our findings suggest that continuous transmission influences the quality rather than simply the magnitude of antigen-experienced memory responses. CT individuals displayed reduced cytokine recall responses together with a persistent CD8^+^CD45RA^−^CCR7^−^ activation-associated phenotype, but without evidence of broad enhancement of proliferative capacity. A dissociation between activation and proliferative capacity has been described in several settings of persistent antigen exposure (32); however, the present study did not assess inhibitory co-receptors or other molecular features required to establish an exhaustion phenotype. Therefore, T-cell exhaustion cannot be inferred from the present data, and the mechanisms underlying the observed activation/proliferation patterns remain unresolved. These findings have implications for VL elimination settings, where declining transmission may influence the durability and quality of immune memory. Understanding immune memory in low-transmission settings is critical, as change in antigen exposure may influence long-term protection and susceptibility to reinfection (4). At the same time, our findings indicate that continuous transmission may also influence immune function with CT individuals displaying a persistent activation-associated phenotype, particularly within CD8^+^CD45RA^−^CCR7^−^ lymphocyte, despite reduced IFN-γ recall responses and limited evidence of enhanced proliferative capacity. Together, these observations highlight the importance of assessing multiple aspects of memory T-cell quality, rather than activation alone, when evaluating immunity in endemic populations.

While this study provides valuable insights into the cytokine profiles and immune memory dynamics in post treated VL, there are certain limitations that should be considered, as the small sample size (n=24) limits the generalizability of our findings.

Additionally, the use of SLA as a complex antigen does not allow identification of epitope-specific responses, and future studies using defined antigen systems may provide greater mechanistic resolution. Functional assays assessing parasite killing or macrophage activation were not performed, which limits direct interpretation of protective immunity.

An important limitation is the substantial age difference among the study groups, with the CT cohort being considerably younger than the NT and OB cohorts. Age is associated with changes in lymphocyte differentiation, baseline activation, cytokine responsiveness, and proliferative capacity and therefore represents an important potential confounder in the present analysis. Because each group contained only eight participants, multivariable adjustment or meaningful age-stratified analysis could not be performed reliably. Consequently, we cannot determine the relative contributions of age and transmission setting to the observed immunological differences, and some or potentially a substantial component of the differences observed in the CT group may reflect its younger age distribution rather than transmission history alone. The findings should therefore be interpreted as unadjusted associations between immunological parameters and transmission setting and not as evidence that transmission intensity independently caused the observed immune phenotypes. In addition, classification into NT, OB, and CT groups was based on the transmission history of the participant’s residential localities rather than individual-level exposure histories. Information on migration, duration of continuous residence, and travel to other VL-endemic areas was not systematically collected. Therefore, exposure outside the assigned residential transmission setting cannot be excluded and represents a potential source of exposure misclassification and residual confounding. Accordingly, the observed immunological differences should be interpreted as associations with area-level transmission setting rather than as definitive consequences of individual antigen-exposure history.

Another limitation of this study is that CD3 was not included in the staining panel. Consequently, although lymphocytes were identified by FSC/SSC properties and memory subsets defined using CD45RA and CCR7, we cannot completely exclude contamination by non-T lymphocyte populations. Accordingly, the observed populations should be interpreted as CD4^+^ or CD8^+^ lymphocyte subsets displaying classical memory phenotypes. In addition, inhibitory co-receptors and molecular markers associated with T-cell exhaustion, including PD-1, LAG-3, CTLA-4, and TIM-3, were not assessed. Consequently, the present study cannot establish whether the observed dissociation between activation, cytokine production, and proliferation represents a true molecular exhaustion phenotype. Any relationship to exhaustion should therefore be considered a hypothesis requiring direct phenotypic and functional validation in future studies. Future studies with larger cohorts are necessary to confirm these results and further explore the role of memory T cells in different transmission settings. Our study’s cross-sectional design provides a snapshot of the immune response at a specific time point. Longitudinal studies tracking immune responses before and after transmission events would provide a more comprehensive understanding of immune memory persistence and reactivation in the context of VL. While we measured Th1, Th2, and Th17 cytokines, the role of other cytokines, such as tumour necrosis factor-alpha and IL-17, in the immune response to VL should also be explored. Additionally, more detailed cytokine profiling using multiplex assays could provide a deeper understanding of the cytokine network involved in immunity. While we observed differences in memory phenotype showing lymphocytes activation, further studies are needed to assess exhaustion markers (e.g., PD-1, LAG-3) in individuals from different transmission settings or transmission intensity to better understand the mechanisms underlying immune differences.

Collectively, these findings suggest that individuals residing in areas with distinct VL transmission histories exhibit differences in post-treatment immune responses following VL cure. These findings support an association between area level transmission setting and qualitative aspect of immune response. However, given the small sample size, potential confounding by difference in age between groups and unmeasured individual mobility and historical exposure, these findings cannot establish an independent causal effect of transmission intensity on immune memory.

## Data Availability

The original contributions presented in the study are included in the article/**Supplementary Material**. Further inquiries can be directed to the corresponding author.
