# Supplementary material for: CD4+ and CD8+ lymphocyte responses and cytokine profiles in visceral leishmaniasis patients following effective treatment in distinct transmission settings in Bihar, India
Source: Front Immunol. 2026 Oct 22;17:1919529. doi: 10.3389/fimmu.2026.1919529 (PMC13715857; doi:10.3389/fimmu.2026.1919529)
Supplement: Supplementary file 1 [file DataSheet1.pdf]

SUPPLEMENTARY DATA

Supplementary Table

Table S1: List of antibodies and reagents

**Flow cytometer antibodies and reagents**			
**S. no.**	**Reagents**	**Company**	**CatLog Number**
1.	CD8-APC	BD Biosciences	570816
2.	CD4-BV605	BD Biosciences	562658
3.	CD45-RA-FITC	BD Biosciences	555488
5.	CCR7-PECy7	BD Biosciences	557648
6.	CD69-BV711	BD Biosciences	563836
7.	Ki67-PE	BD Biosciences	570923
8.	Zombie Aqua	BioLegend	423102
9.	Anti-CD3	BD Biosciences	550368
10.	Anti-CD28	BD Biosciences	555726
11.	BD Cytofix/Cytoperm	BD Biosciences	512090KZ
12.	BD Perm/Wash	BD Biosciences	512091KZ
**Other Major Reagents**			
13.	Phosphate-Buffered Saline (1X)	Gibco (ThermoFisher Scientific)	10010023
14.	RPMI-1640 (1X)	Gibco (ThermoFisher Scientific)	22400089
15.	Heat-Inactivated Fetal Bovine Serum (HI-FBS)	Gibco (ThermoFisher Scientific)	10082147

16.	Lymphoprep	STEMCELL Technologies	18061
17.	Phytohemagglutinin	Sigma-Aldrich (Merck)	L1668
18.	Penicillin-Streptomycin	Gibco (ThermoFisher Scientific)	15140122
19.	Polypropylene round-bottom tubes (5 ml)	Falcon (CORNING)	352063
20.	Polystyrene round-bottom tubes (5 ml)	BD Falcon (BD Biosciences)	352054
21.	Trypan Blue Solution	Sigma-Aldrich (Merck)	T8154
22.	Human Th1/Th2/Th17 Cytokine Kit (CBA Kit)	BD Biosciences	560484

Supplementary Figures

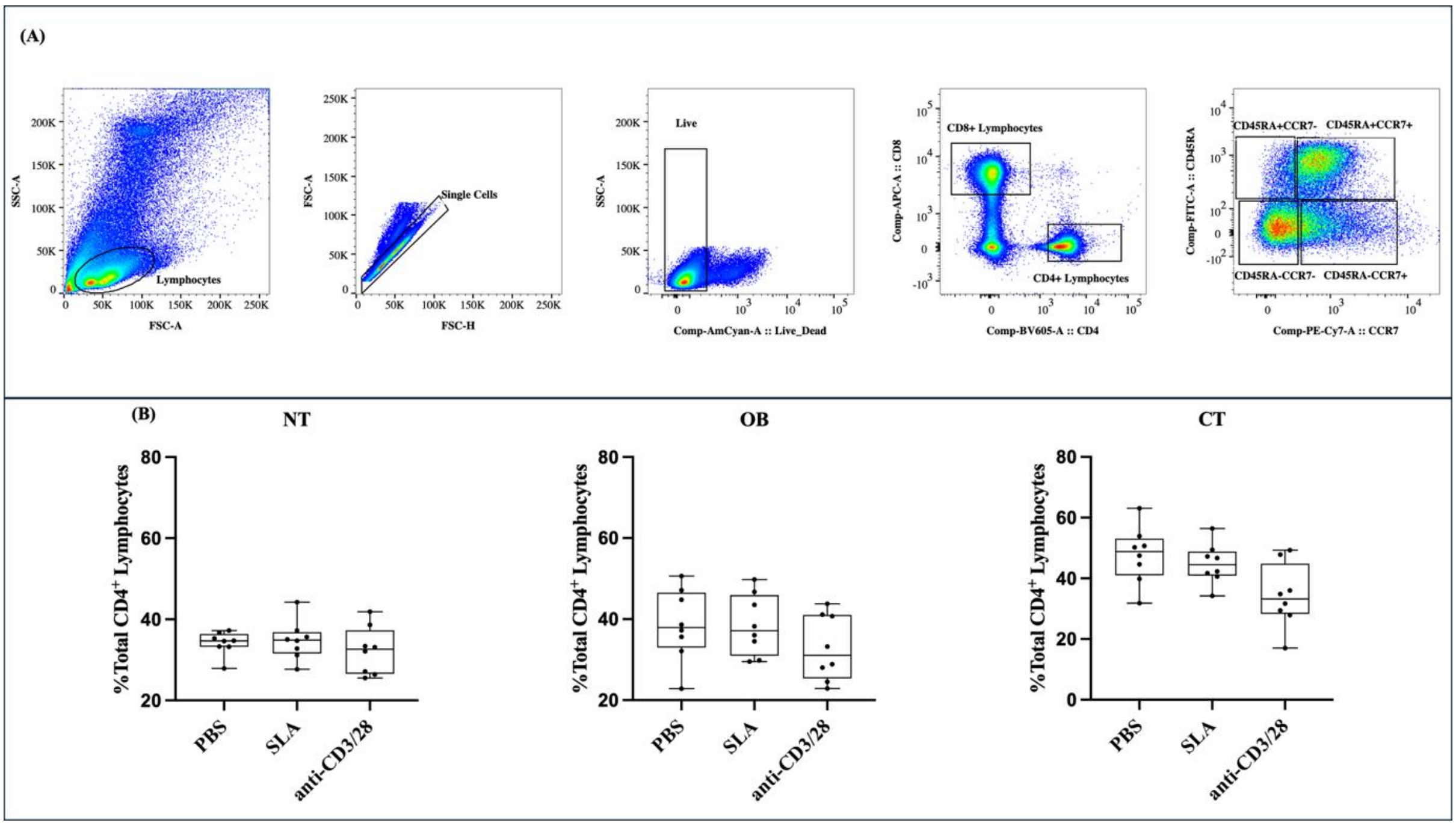

Figure S1: Representative flow cytometry gating strategy used for identification of CD4⁺ and CD8⁺ lymphocyte populations and CCR7/CD45RA-defined differentiation phenotypes and total CD4⁺ lymphocyte frequency.

Cells were first gated based on lymphocyte characteristics using forward scatter area (FSC-A) and side scatter area (SSC-A), followed by singlet discrimination using FSC-A versus FSC-H. Live cells were subsequently identified by exclusion of Zombie Aqua viability dye-positive cells. The live lymphocyte population was then separated into $CD4^+$ and $CD8^+$ lymphocyte populations, followed by characterization based on CD45RA and CCR7 expression. Naïve ($CD45RA^+CCR7^+$) and $CD45RA^-CCR7^+$, $CD45RA^-CCR7^-$ and $CD45RA^+CCR7^-$ memory-like lymphocyte populations were identified within $CD4^+$ and $CD8^+$ compartments. As CD3 was not included in the acquired panel, these populations are described according to CD4/CD8 and CD45RA/CCR7 expression profiles. Representative gating strategy is shown in (A). Total $CD4^+$ lymphocyte frequencies following soluble Leishmania antigen (SLA) stimulation compared with NIL (PBS)

across NT, CT and OB groups are shown in (B). Statistical analysis was performed using the Wilcoxon matched-pairs signed-rank test.

Abbreviations: FSC-A, forward scatter area; FSC-H, forward scatter height; CCR7, C-C chemokine receptor type 7; CD45RA, CD45 isoform RA; SLA, soluble Leishmania antigen; PBS, phosphate-buffered saline; NT, no transmission; OB, outbreak; CT, continuous transmission.

.

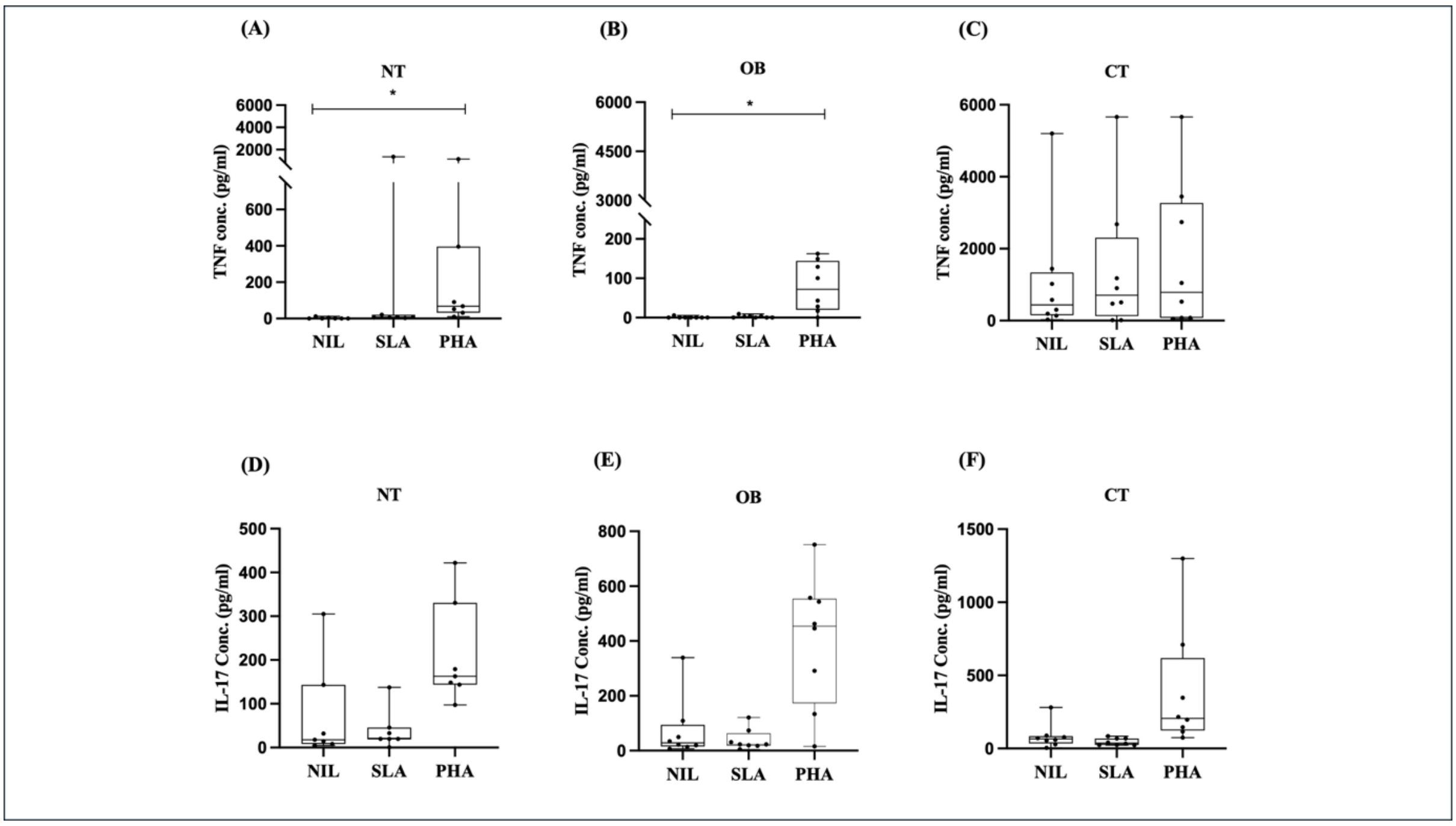

Figure S2: Additional cytokine recall responses following SLA stimulation

Additional cytokine responses following SLA stimulation in individuals from NT, OB, and CT groups are shown. TNF-α (A–C) and IL-17 (D-F) responses following SLA stimulation are shown across NT, OB, and CT groups. Cytokine responses were assessed following SLA stimulation and compared with unstimulated controls (PBS/NIL) within individuals. Statistical comparisons between stimulation conditions were performed using the Wilcoxon matched-pairs signed-rank test. Data represent cytokine concentrations measured following stimulation.

Abbreviations: SLA, soluble *Leishmania* antigen; PBS, phosphate-buffered saline; NIL, unstimulated control; TNF-α, tumor necrosis factor-alpha; IL, interleukin; NT, no transmission; OB, outbreak; CT, continuous transmission. Statistical significance was defined as *p < 0.05 and **p < 0.01.

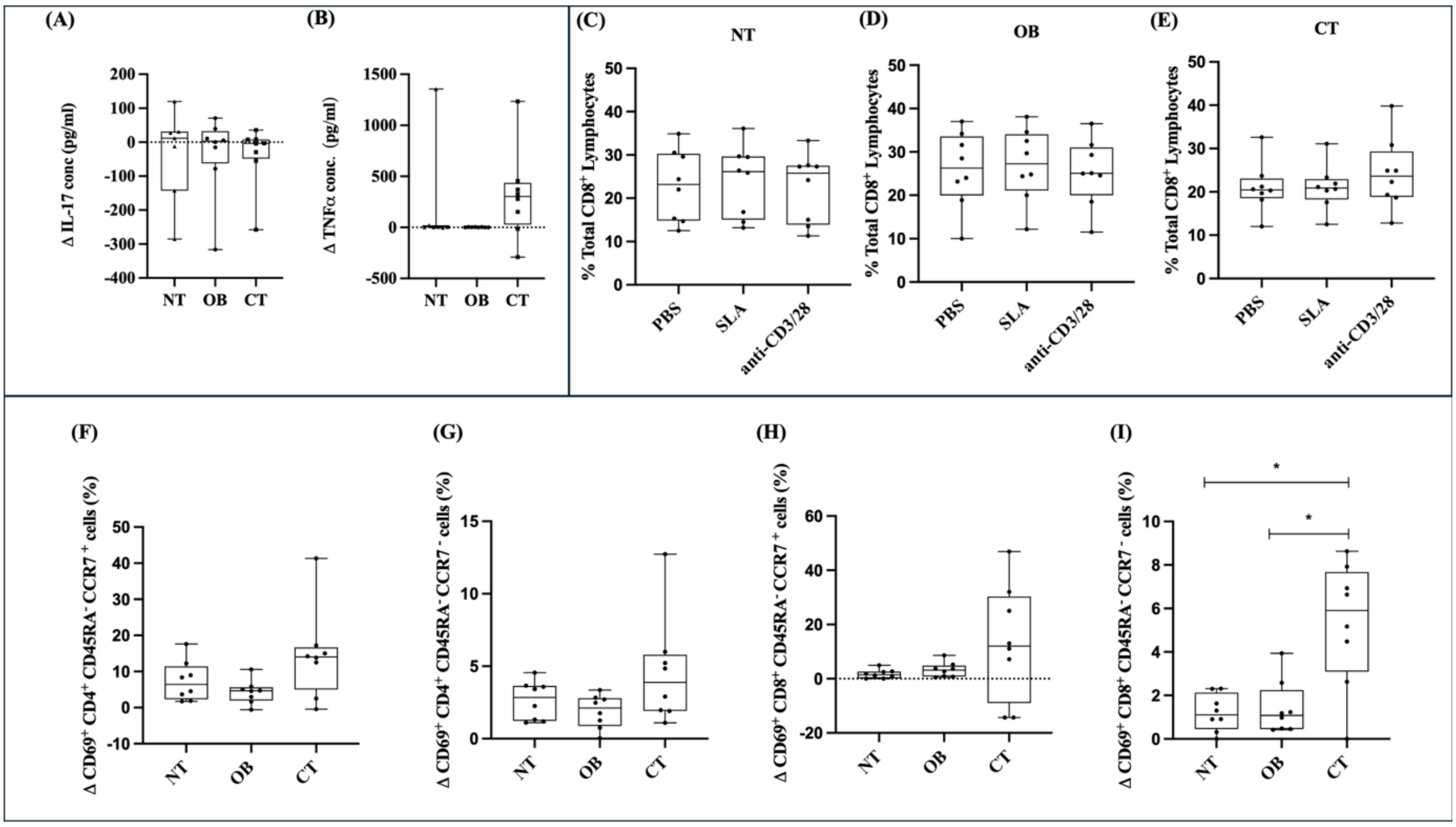

Figure S3: Cytokine recall responses, total CD8$^+$ lymphocyte frequencies, and baseline-normalized CD69 activation responses within CCR7/CD45RA-defined lymphocyte populations following SLA stimulation.

ΔIL-17 and ΔTNF-α responses following SLA stimulation across NT (n=7), OB (n=8), and CT (n=8) groups are shown in (A–B). Δ cytokine responses were calculated as SLA-induced cytokine production above unstimulated control values (Δ = SLA − PBS/NIL). Total CD8$^+$ lymphocyte frequencies under PBS, SLA, and anti-CD3/CD28 stimulation conditions across NT (n=8), OB (n=8), and CT (n=8) groups are shown in (C–E). PBS and anti-CD3/CD28 stimulation were included as negative and positive control conditions, respectively.

Antigen-induced CD69 activation responses were calculated as ΔCD69 (SLA−PBS) within CD4$^+$ CD45RA$^-$CCR7$^+$ (F), CD4$^+$CD45RA$^-$CCR7$^-$ (G), CD8$^+$CD45RA$^-$CCR7$^+$ (H), and CD8$^+$

$CD45RA^{-}CCR7^{-}$ (I) lymphocyte populations across NT, OB, and CT groups. Following baseline normalization, no significant differences were observed among $CD4^{+}CD45RA^{-}CCR7^{+}$ $CD4^{+}CD45RA^{-}CCR7^{-}$, or $CD8^{+}CD45RA^{-}CCR7^{+}$ lymphocyte populations. $CD8^{+}CD45RA^{-}CCR7^{-}$ lymphocyte populations showed a significant overall difference among groups (Kruskal–Wallis $p = 0.0142$), with higher ΔCD69 responses in CT compared with NT (adjusted $p = 0.0266$) and OB (adjusted $p = 0.0463$), while NT and OB did not differ significantly. Statistical comparisons were performed using the Wilcoxon matched-pairs signed-rank test for paired stimulation conditions and Kruskal–Wallis test with Dunn's multiple comparison correction for between-group comparisons.

Abbreviations: Δ, antigen-induced change; CD69, activation marker; CCR7, C-C chemokine receptor type 7; CD45RA, CD45 isoform RA; SLA, soluble *Leishmania* antigen; PBS, phosphate-buffered saline; NT, no transmission; OB, outbreak; CT, continuous transmission.

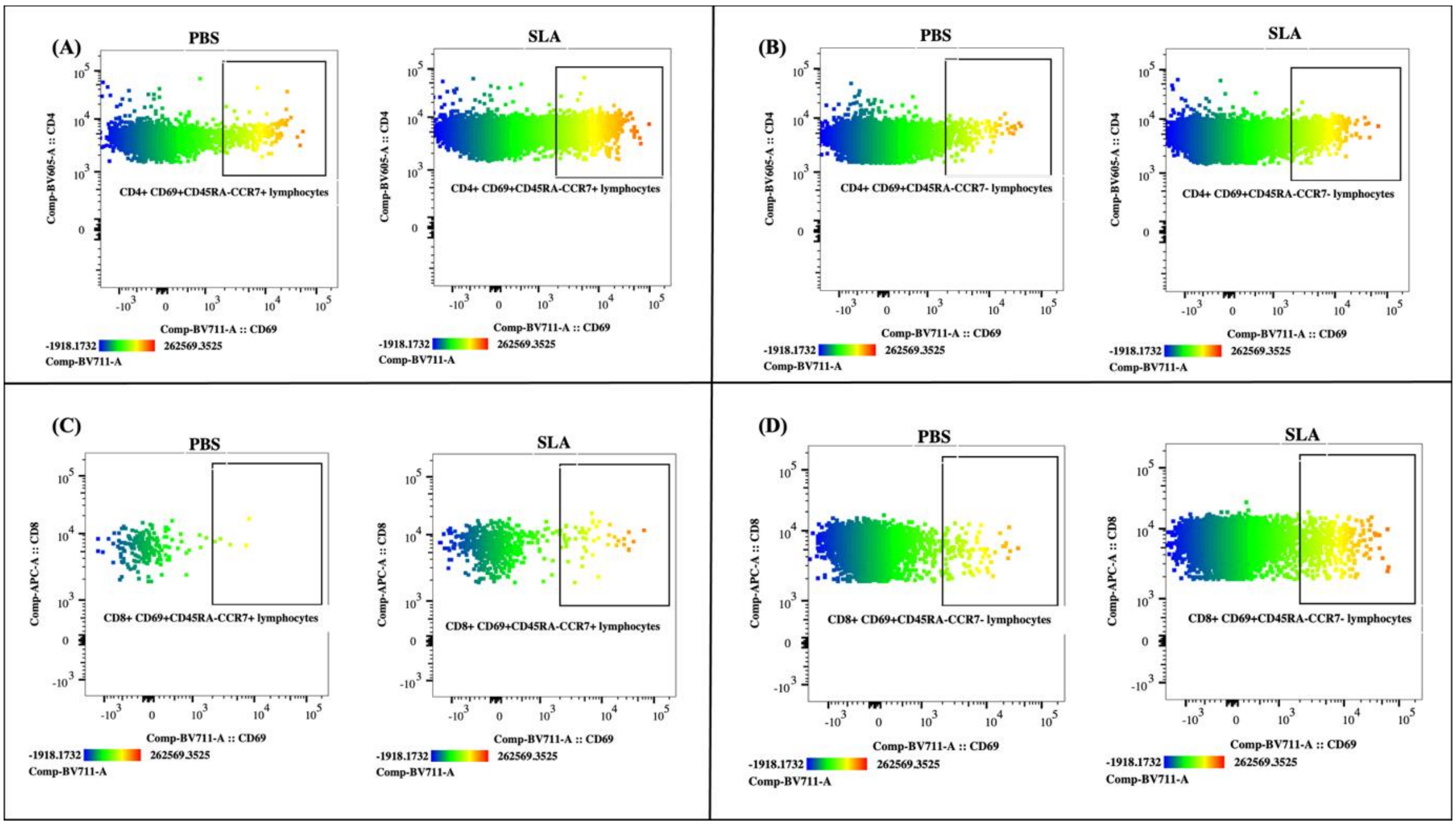

Figure S4: Representative FlowJo heatmap analysis of CD69 expression within $CD4^{+}$ and $CD8^{+}$ CCR7/CD45RA-defined lymphocyte populations following SLA stimulation.

Representative bivariate flow cytometry plots with FlowJo heatmap statistics showing CD69 expression within CD4⁺ CD45RA⁻ CCR7⁺ lymphocyte populations (A), CD4⁺ CD45RA⁻ CCR7⁻ lymphocyte populations (B), CD8⁺ CD45RA⁻CCR7⁺ lymphocyte populations (C), and CD8⁺ CD45RA⁻CCR7⁻ lymphocyte populations (D) following PBS and SLA stimulation. The same gating strategy and CCR7/CD45RA-defined lymphocyte populations used for quantification in Figures 3–5 were applied for representative visualization. Populations were identified based on CD45RA and CCR7 expression, followed by assessment of CD69 expression within the respective gated populations. Heatmap statistics generated using FlowJo represent the frequency and distribution of CD69⁺ cells within each analysed population.

Abbreviations: CD69, activation marker; CCR7, C-C chemokine receptor type 7; CD45RA, CD45 isoform RA; SLA, soluble *Leishmania* antigen; FlowJo, flow cytometry analysis software.

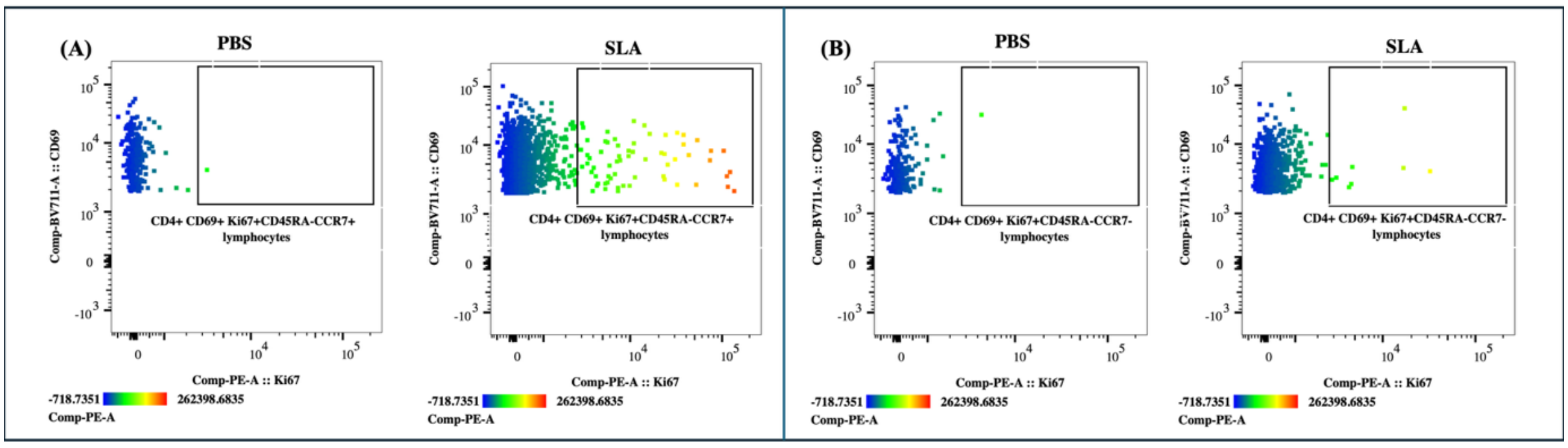

Figure S5: Representative FlowJo heatmap analysis of Ki67 expression within CD69⁺ activated CD4⁺ CCR7/CD45RA-defined lymphocyte populations.

Representative bivariate flow cytometry plots with FlowJo heatmap statistics showing Ki67 expression within CD69⁺ activated CD4⁺CD45RA⁻CCR7⁺ lymphocyte populations (A) and CD69⁺ activated CD4⁺CD45RA⁻CCR7⁻ lymphocyte populations (B) following soluble Leishmania antigen (SLA) stimulation. The same gating strategy and analysed populations

used for quantification in Figure 6 were applied for representative visualization. $CD4^+CD45RA^-CCR7^+$ and $CD4^+CD45RA^-CCR7^-$ lymphocyte populations were identified based on CD45RA and CCR7 expression, followed by assessment of Ki67 expression within $CD69^+$ activated populations. Heatmap statistics generated using FlowJo represent the frequency and distribution of $Ki67^+$ cells within the indicated $CD69^+$ activated lymphocyte populations.

Abbreviations: Ki67, proliferation-associated nuclear marker; CD69, activation marker; CCR7, C-C chemokine receptor type 7; CD45RA, CD45 isoform RA; SLA, soluble *Leishmania antigen*; FlowJo, flow cytometry analysis software.
